# Tuberculosis cure rates and the ETR.Net: investigating the quality of reporting treatment outcomes from primary healthcare facilities in Mpumalanga province, South Africa

**DOI:** 10.1186/s12913-017-2128-0

**Published:** 2017-03-10

**Authors:** A. W. Dreyer, D. Mbambo, M. Machaba, C. E. M. Oliphant, M. M. Claassens

**Affiliations:** 1 Department of Medical Microbiology, University of Pretoria and the National Health Laboratory Services (NHLS), Pretoria, South Africa; 2 Centre for Tuberculosis, National Institute for Communicable Diseases, 1 Modderfontein Road, Sandringham, South Africa; 3 Department of Health, Mpumalanga Province, Mpumalanga, South Africa; 4 Desmond Tutu TB Centre, Department of Paediatrics and Child Health, Stellenbosch University, Cape Town, South Africa

**Keywords:** Quality of data, ETR.Net, Treatment outcome

## Abstract

**Background:**

Tuberculosis control programs rely on accurate collection of routine surveillance data to inform program decisions including resource allocation and specific interventions. The electronic TB register (ETR.Net) is dependent on accurate data transcription from both paperbased clinical records and registers at the facilities to report treatment outcome data. The study describes the quality of reporting of TB treatment outcomes from facilities in the Ehlanzeni District, Mpumalanga Province.

**Methods:**

A descriptive crossectional study of primary healthcare facilities in the district for the period 1 January – 31 December 2010 was performed.

New smear positive TB cure rate data was obtained from the ETR.Net followed by verification of paperbased clinical records, both TB folders and the TB register, of 20% of all new smear positive cases across the district for correct reporting to the ETR.Net. Facilities were grouped according to high (>70%) and low cure rates (≤ 70%) as well as high (> 20%) and low (≤ 20%) error proportions in reporting. Kappa statistic was used to determine agreement between paperbased record, TB register and ETR.Net.

**Results:**

Of the100 facilities (951 patient clinical records), 51(51%) had high cure rates and high error proportions, 14(14%) had a high cure rate and low error proportion whereas 30(30%) had low cure rates and high error proportions and five (5%) had a low cure rate with low error proportion. Fair agreement was observed (Kappa = 0.33) overall and between registers. Of the 473 patient clinical records which indicated cured, 383(81%) was correctly captured onto the ETR.Net, whereas 51(10.8%) was incorrectly captured and 39(8.2%) was not captured at all. Over reporting of treatment success of 12% occurred on the ETR.Net.

**Conclusions:**

The high error proportion in reporting onto the ETR.Net could result in a false sense of improvement in the TB control programme in the Ehlanzeni district.

## Background

Tuberculosis (TB) is a global public health threat with an incidence of 9.6 million cases and 1.3 million deaths reported in 2014 [1]. However, a dramatic reduction in TB incidence as well as death rates has been observed in some countries. The prevalence rate of TB has declined globally by 42% from 1990 to 2014, with a 50% reduction in three World Health Organization (WHO) regions and nine of the high-burdened countries, and is most likely the result of nationwide prevalence surveys, adequate sample registration systems and electronic case notification [1]. The performance of TB treatment programs are measured against notification rates as well as successful treatment outcomes of new smear positive cases. Poor performing countries that are not able to reach the benchmark of 85% are monitored for overall improvement in performance. In 2014 the estimated global notification rate was 63% with a global treatment success rate of 86% in 2014 [1].

TB incidence rates in South Africa remain high and range from 737 to 936 cases per 100 000 population with 318 193 cases notified in 2014 [1]. The electronic TB register (ETR.Net) was introduced in 2004 to improve the collection and utilization of TB data across South Africa [2]. Other countries followed and successfully implemented the system including Botswana, Guatemala, Mozambique, Namibia, Swaziland and Tanzania [2]. It is a standardized computer based software program that can be utilized from national down to sub-district level. This system allows for electronic capturing of patient-level information from paperbased TB registers at the district level for submission to national level. In addition, standard cohort reports and line listing data can be generated which enable staff to monitor and evaluate the TB program. This is achieved by the generation of monthly and quarterly reports pertaining to TB treatment outcomes which are based on the information entered into the system, for instance cure rates, completion rates, defaulter rates, death rates, patients transferred out and data not evaluated. The system relies on the accurate reporting of information from the patient clinical records and TB registers into the electronic system.

In South Africa, new smear positive patients are treated with combination therapy for 6 months (2 months intensive phase with rifampicin, isoniazid, pyrazinamide and ethambutol and 4 months continuation phase with rifampicin and isoniazid). Patients with documented smear and or culture conversion at the end of treatment are recorded as cured. Treatment outcomes are recorded in the patient clinical record and transcribed to the tuberculosis register by primary healthcare givers at the facilities. The district TB coordinator collects on a weekly basis a copy of the completed register and captures this information on the ETR.Net. Updated information (reporting) is provided to provincial managers monthly and to the national program quarterly to determine treatment outcome estimates. Subsequently this data is summarized and annually reported to the WHO. The quality of data recorded into the electronic register is not often investigated, especially when areas are performing well, for example have high reported cure rates. In the Mpumalanga province, the number of multidrug resistant TB (MDR-TB) cases within the three districts has doubled from 190 in 2009 to 385 in 2011 [3]. In the Ehlanzeni district good treatment outcomes (cure rates of >79%) were reported despite the high prevalence of MDR-TB of which 159 cases were reported for 2010. In comparison, the other districts had cure rates of 64 and 68% with lower numbers of MDR-TB cases [4]. Whether these reported cure rates are accurate, the reporting system of the TB control program is functioning optimally and the reports are a true reflection of the province’s TB management should be answered. With a functioning TB control program cure rates are expected to be high with low numbers of MDR-TB and vice versa [5], however high MDR-TB rates within the province have been confirmed in other studies [6, 7] despite the high cure rates.

Previous studies from South Africa have investigated the accuracy of case registration. A Western Cape study reported 17% of smear positive cases not being reported and started on treatment [8] while studies performed in Gauteng and Kwazulu-Natal reported around 20% of cases not being recorded in the TB register [9, 10]. Other studies found that the accuracy and completeness of data in the TB treatment register linked with the central laboratory data were inadequate and that a high number of bacteriologically confirmed cases were not included in the TB register [11, 12]. A recent study in 2015 reported on a national evaluation of the ETR.net for a period in 2009 and found that not all TB diagnosed patients were captured and the completeness and reliability of the electronic data was inconsistent across different data sources [13]. Incorrect reporting of TB treatment outcomes at facilities can lead to inaccurate reporting of district or provincial cure rate data. The aim of this study was to describe the quality of TB case management reporting among primary healthcare facilities (PHC facilities) in the Ehlanzeni District, Mpumalanga province.

## Methods

### Study setting and design

The study was conducted at all primary healthcare facilities (PHC) facilities rendering TB management services within the Ehlanzeni District, Mpumalanga province, South Africa. The study was a descriptive cross-sectional study of the PHC facilities within the district where a random 20% selection of new smear positive (NSP) cases during the period 1 January 2010 to 31 December 2010 were included.

### Data variables, data source and data collection methods

**Fig. 1:**
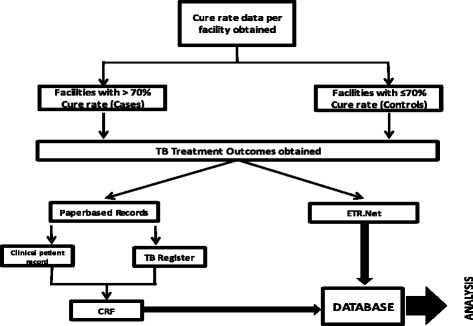
Methodology: Cure rate data obtained and facilities divided into cases and controls. Patient clinical records captured with CRF and entered into database. Treatment outcomes obtained from ETR.Net and entered into database. Patient clinical records verified against electronic register for correct reporting of treatment outcomes

The study investigated PHC facilities which were grouped according to cure rate data that were electronically obtained from the ETR.Net. Field workers were employed through funding to visit selected facilities and verify the paperbased clinical records, both the TB folders and the TB register, of 20% of all new smear positive cases selected randomly at every facility across the district for the correct reporting of the TB treatment outcomes in the ETR.Net. Facilities were excluded when they reported low numbers (< 15) NSP cases for study period and also if the records were not accessible. Paperbased data were captured with a case record form (CRF) followed by entry into a database and manual correlation with ETR.Net data (Fig. 1). Variables captured included an end of treatment smear result and as well as the respective treatment outcome.

### Study definitions

Reported cure rates on the ETR.Net of >70% and ≤ 70% were considered as high and low respectively, the cutoff was decided based on the national treatment success rate of >70% for the last 5 years (2005–2010) [1]. Reporting of standard TB treatment outcomes as defined according to the WHO (cured, completed, died, failed, defaulted and transferred out) [14] were used to determine error. Primary error was defined as any disagreement at the paperbased level (patient clinical record vs. paperbased TB register) and secondary error was any disagreement between the paperbased TB register and the ETR.Net. Total error was defined as any disagreement at any level. For instance primary error was calculated as the number of patient clinical records where the treatment outcomes were incorrectly captured in the TB register divided by the total number of records reviewed (20%) and expressed as a percentage. Error proportions per facility were defined as high (> 20%) and low (≤ 20%) and calculated as the total number of disagreement (Total error) at any level over the amount of clinical records viewed. The cutoff for error (20%) was based on a potential error rate of 5–20% that can occur when processing data as previously reported as well as a consensus decision among investigators of a reasonable error rate (two out of every ten records per facility) that can occur within an operational surveillance setting [15].

### Statistical analysis

Means were calculated for primary, secondary and total error rates across all facilities. The total number of clinical records not captured in ETR.Net as well as the availability of the end of treatment smear results was determined. A cross population table was drawn for the correlation of treatment outcomes from patient clinical records and ETR.Net. The kappa statistic was performed to assess the overall level of agreement between patient clinical record vs. paperbased TB register and paperbased TB register and the ETR.Net across all investigated facilities within the district. Sub-analysis was performed to assess the difference among facilities with high and low reported cure rates.

## Results

**Fig. 2:**
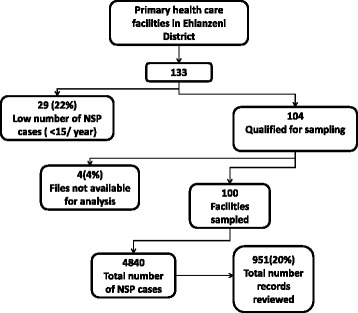
Selection of facilities for sampling

Of the 133 primary healthcare facilities in the province, 29 were excluded due to low numbers of NSP cases. A further four were excluded due to inaccessibility of clinical records. A total number of 951 records were verified across 100 facilities which represents 20% of NSP (*N* = 4840) cases from included facilities (Fig. 2). The average patient clinical records reviewed per facility was 10 (range 3–58). The four facilities excluded represented 28 records.

The median cure rate across all facilities was calculated at 75%. Sixty-five (65%) and 35 (35%) facilities were classified with high and low cure rates respectively. Primary error was observed among 245 (25.8%) records, secondary error among 316 (33.2%), both primary and secondary error 117 (12.3%) and total error among 444 (46.7%). The kappa agreements between the paperbased clinical record and the papaerbased TB register were 0.26 (overall facilities), 0.24 (high cure rate facilities) and 0.30 (low cure rate facilities). The kappa agreements between the paperbased TB register and the ETR.Net were 0.33 (overall facilities), 0.33 (high cure rate facilities) and 0.34 (low cure rate facilities).

Fifty-one facilities (51%) had high cure rates and high error proportions, 14 (14%) had high cure rates with low error proportions, while 30 (30%) had low cure rates and high error proportions and 5 (5%) low cure rates and low error proportions (Table 1). The correlation of TB treatment outcomes is shown in Table 2.

**Table 1:** Percentage of facilities grouped according to cure rate and error reporting (*N* = 100)

		Outcome: Facility Cure rate (%)		
		High (> 70%)	Low (≤ 70%)	Totals
Determinant: Error reporting (%)	High (> 20%)	51	30	71
	Low (≤ 20%)	14	5	19
	Totals	65	35

**Table 2:** Cross population table of TB treatment outcomes correlation between the patient clinical records and the electronic TB register across Ehlanzeni District, Mpumalanga (2010) *N* = 951

		ETR.Net							
		Cured	Completed	Failure	Died	Transferred	Defaulted	Not recorded	Total
Patient clinical record	Cured	383^a^	43^b^	0	3^b^	3^b^	2^b^	39^c^	473
	Completed	57	54	0	2	1	0	17	131
	Failure	1	1	4	0	1	0	1	8
	Died	8	1	1	30	0	0	10	50
	Transferred	6	6	1	0	61	0	6	80
	Defaulted	14	5	1	2	4	21	13	60
	Not evaluated	77	20	2	10	11	7	22	149
	Total	546	130	9	47	81	30	108	951

^a^Total number of cured cases correctly reported (383/473)

^b^Total number of cured cases reported incorrectly (as other outcomes e.g. cured as died) (39/473)

^c^Total number of cured cases not captured in ETR.NET (39/473)

Of the 473 patient clinical records which indicated cured, 383 (81%) was correctly captured into the electronic TB register, whereas 51 (10.8%) was incorrectly captured and 39 (8.2%) was not captured at all. Eighty-six (9%) of records were incorrectly captured as cured. Seventy-seven records (8%) were not evaluated on the patient clinical records but captured electronically as cured.

Fourty-three (4.5%) patient clinical records indicating cured were incorrectly captured in the ETR.net as completed and 57 (6%) records indicating completed was incorrectly captured as cured. Almost 10% of all records were incorrectly captured as cured which included patients who have died (*N* = 8), had treatment failure (*N* = 1), defaulted treatment (*N* = 14) and were transferred out of the facilities (*N* = 6). Of the 951 records 604 had either completed or cured correctly in ETR.Net indicating treatment success, however an over report of cured and completed of 12% on the electronic register occurred. Death and defaulted as outcomes were poorly reported in 60% (30/50 cases) and 54% (21/39 cases) respectively.

Of the 951 clinical records verified, 108 (11.4%) were not captured into the ETR.Net. In 337 (39.6%) reviewed records there was absence of documented end of treatment smear results.

## Discussion

Accurate reporting of TB treatment outcomes especially cure rate is critical in understanding a rise in MDR-TB which could be related to treatment failure or a high defaulter rate and assisting national programs in optimizing strategies to improve TB control. Our study reports on the quality of the treatment outcome data of the ETR.Net across the Ehlanzeni district in Mpumalanga province.

The study included a representative sample of facilities within the district. The observed primary (patient clinical record to paperbased TB register) and secondary (paperbased TB register to ETR.Net) errors are of concern as this contributes directly to incorrect reporting in the electronic register. The agreement statistics performed revealed all within the fair agreement range (0.21–0.40) [16]. Reasons for these errors could be transcription related, negligence, misinterpretation and/or the unavailability of paperbased records despite ongoing pressure to complete the electronic register in time for compilation of national statistics. These need to be further investigated. Despite training being provided at facility level, the method is not standardized and competency not assessed across all facilities. The high demand on providing primary health services may compromise effective data reporting. Although the study forms a formalized quality assessment, realtime quality control procedures (re-checking system) for data entry needs to be put in place to ensure accurate reporting.

Of concern is the high overall error at any level that was observed across all facilities. With regard to cure rate we observed > 80% of patient clinical records correctly captured, however the remaining records that were incorrectly or not captured could affect the overall cure rate data. Another contributing factor could be that almost 10% of all records were incorrectly captured as cured which included patients who have died, had treatment failure or defaulted. Another problem encountered was the 77 records (8%) that were captured as cured but were not evaluated at facility level. This clearly indicates the various levels of error affecting the quality of the cure rate data. Over reporting of treatment success was evident from our study findings. Also accurately reporting of death and defaulting as outcomes was poor. This clearly highlights deficiencies and the potential impact on overall provincial statistics.

More than 10% of record outcomes were incorrectly captured as either completed whilst being cured or vice versa. This highlights the potential problem of misunderstanding the treatment outcome definitions and applying these definitions to patient management. “Cured” is considered with documented confirmation (smear negative) of absence of disease whilst “completed” lacks the evidence. The practice of documentation of end of treatment smear results enforces health care workers to apply the correct definition, however our study showed an absence of end of treatment smear results of almost 40%. Another study by Dilraj et al found an absence of 53.2% of end of treatment smear results in Kwazulu-Natal Province and highlighted the uncertainty with regard to treatment outcome data [17].

Our study also showed the treatment outcome data for 11% of patient clinical records were not captured in the electronic register. A study performed among children with tuberculosis reported only 62% of patients were registered in the ETR.Net and highlighted the underreporting of the burden of disease especially as patients with TB meningitis and death were less likely to be referred and captured on the system [18].

Although we observed more facilities with reported high cure rates and high error proportions we could not demonstrate an association and vice versa. High cure rates would however represent good performing facilities and are more often praised for performance than scrutinized for accuracy of reporting compared to facilities with low reported cure rates. This could influence the rate of reporting by shifting the focus on performance rather than accuracy. Tuberculosis control programs should focus equally on achieving necessary targets (high cure rates) and accuracy of data reporting.

Limitations of the study included the low number of facilities with low cure rates. Although we accurately sampled at district level, our findings may not reflect the overall picture of the province or the country. Also the study was able to identify where the errors occurred but not the reasons behind these errors and further differences among facilities needs to be investigated. Strengths of our study included the ability to correlate recording of treatment outcome data across an appropriately sampled district. Our study also highlights various aspects of the TB control program in South Africa that can be improved or re-inforced e.g. accurately reporting from the patient clinical record to the paperbased TB register and into the electronic register, documentation of the end of smear results and applying the correct definition to improve patient management as well as the quality of the data.

## Conclusions

The high error proportion in reporting on the ETR.Net could result in a false sense of improvement in the TB control programme in the Ehlanzeni district. Quality of data should be verified across all provinces in South Africa to ensure an accurate reflection of improvements in the national TB control programme.

## Abbreviations

CRF: Case record form
ETR.Net: Electronic tuberculosis register
MDR-TB: Multidrug resistant tuberculosis
NSP: New smear positive
PHC: Primary health care
TB: Tuberculosis
WHO: World Health Organization

## References

[1] World Health Organization. Global Tuberculosis control 2015. http://apps.who.int/iris/bitstream/10665/191102/1/9789241565059_eng.pdf. Accessed 11 May 2016.

[2] ETR.Net 2007. http://www.etrnet.info/CountryImplementations.aspx. Accessed 11 May 2016.

[3] Department of Health South Africa. Mpumalanga Tuberculosis Program. Multidrug resistant tuberculosis rates. Electronic Drug Resistance register (EDR.Net). http://www.mpuhealth.gov.za. Accessed 5 Apr 2013.

[4] Department of Health South Africa. Mpumalanga Tuberculosis Program, Treatment Outcome Summary by Unit, New TB Cases, Quarter 1- 4 of 2011. Electronic Tuberculosis register (ETR.NET). http://www.mpuhealth.gov.za. Accessed 5 Apr 2013.

[5] Loddenkemper R, Sagebiel D, Brendel A (2002). Strategies against multidrug-resistant tuberculosis. Eur Respir J.

[6] Green E, Obi CL, Nchabeleng M, Villiers BED, Sein PP, Letsoalo T (2010). Drug-susceptibility patterns of mycobacterium tuberculosis in Mpumalanga Province, South Africa: Possible Guiding Design of Retreatment Regimen. J Health Popul Nutr.

[7] Weyer K, Lancaster J, Balt E, Durrheim D (1998). Tuberculosis drug resistance in Mpumalanga province, South Africa. Int J Tuberc Lung Dis.

[8] Botha E, den Boon S, Lawrence KA (2008). From suspect to patient: tuberculosis diagnosis and treatment initiation in health facilities in South Africa. Int J Tuberc Lung Dis.

[9] Edginton ME, Wong ML, Phofa R, Mahlaba D, Hodkinson HJ (2005). Tuberculosis at Chris Hani Baragwanath Hospital: numbers of patients diagnosed and outcomes of referrals to district clinics. Int J Tuberc Lung Dis.

[10] Bristow CC, Dilraj A, Margot B, Podewils LJ (2013). Lack of patient registration in the electronic TB register for sputum smear-positive patients in KwaZulu-Natal, South Africa. Tuberculosis.

[11] Dunbar R, Lawrence K, Verver S, Enarson DA, Lombard C, Hargrove J (2011). Accuracy and completeness of recording of confirmed tuberculosis in two South African communities. Int J Tuberc Lung Dis.

[12] Mngomezulu N, Cameron D, Olorunju S, Luthuli T, Dunbar R, Naidoo P (2015). Reasons for the low bacteriological coverage of tuberculosis reported in Mpumalanga Province, South Africa. Pub Health Act.

[13] Podewils LJ, Bantubani N, Bristow C, Bronner LE, Peters A, Pym A, Mametja LD (2015). Completeness and reliability of the republic of South Africa National Tuberculosis (TB) Surveillance System. BMC Public Health.

[14] World Health Organization. Tuberculosis (TB) Definitions of treatment outcomes 2014. http://apps.who.int/iris/bitstream/10665/79199/1/9789241505345_eng.pdf. Accessed 11 May 2016

[15] Biemer PP, Lyberg LE, Biemer PP, Lyberg LE (2003). Data processing: Errors and their control. Introduction to survey quality.

[16] Veira AJ, Garrett JM (2005). Understanding interobserver agreement: the Kappa statistic. Fam Med.

[17] Dilraj A, Bristow CC, Conolly C, Margot B, Dlamini S, Podewils LG (2013). Validation of sputum smear results in the Electronic TB Register for the management of tuberculosis, South Africa. Int J Tuberc Lung Dis.

[18] Du Preez K, Schaaf HS, Dunbar R, Swartz A, Bissel K, Enarson DA, Hesseling AC (2011). Incomplete registration and reporting of culture-confirmed childhood tuberculosis diagnosed in hospital. Pub Health Act.

